# Cauda Equina Syndrome Due to Spinal Subdural Hematoma Mimicking Epidural Hematoma: The “Tense Bluish Dura” Intraoperative Clue

**DOI:** 10.7759/cureus.113446

**Published:** 2026-07-27

**Authors:** Bashar Altunbi, Oludare Ashaolu, Lubna Rahim, Navin Verghese

**Affiliations:** 1 Spinal Surgery, Swansea Bay University Health Board, Swansea, GBR; 2 Trauma and Orthopaedics, Swansea Bay University Health Board, Swansea, GBR; 3 Spinal Surgery, Swansea Bay University Health Board, swansea, GBR

**Keywords:** cauda equine syndrome, laminectomy, magnetic resonance imaging, oral anticoagulation, spontanous spinal subdural hematoma

## Abstract

Spontaneous spinal subdural hematoma is an uncommon cause of acute cauda equina syndrome that can closely resemble spinal epidural hematoma on magnetic resonance imaging (MRI). This report describes an intraoperative dural appearance that may help identify subdural pathology when imaging suggests an epidural source. A 73-year-old woman receiving apixaban for recurrent pulmonary embolism presented with sudden lumbar pain radiating to both legs, followed by right-sided leg weakness and urinary retention. MRI showed a dorsal collection from L1 to L3 reported as an epidural hematoma, with a right L1-L2 facet joint cyst supporting this interpretation. She underwent urgent L1-L4 laminectomy. After epidural decompression and facet cyst removal, the dura remained tense, nonpulsatile, and bluish. This appearance prompted a midline durotomy, which revealed an intact arachnoid and a dark, organizing subdural hematoma. The clot was evacuated, and the cauda equina was decompressed. Radicular pain resolved immediately. At two weeks, the left leg had fully recovered, and the right leg showed partial improvement. At three months, she was walking independently with full neurological recovery. A brief literature review identified similar intraoperative descriptions of dural discoloration and persistent tension when imaging did not clearly distinguish epidural from subdural collections. A tense, nonpulsatile, bluish dura after epidural decompression may indicate an underlying subdural hematoma. Recognition of this appearance can guide dural exploration, especially in anticoagulated patients with additional findings that may influence MRI interpretation.

## Introduction

Cauda equina syndrome (CES) is a surgical emergency requiring urgent decompression. The differential diagnosis is broad and includes disc herniation, spinal stenosis, infection, vascular malformations, and spontaneous spinal hemorrhage [1,2]. Among hemorrhagic causes, epidural hematoma is most frequently encountered, followed by the rarer subdural hematoma [2]. Distinguishing these entities on MRI is often unreliable [1], as both may appear as crescentic or biconvex dorsal collections. Anticoagulation is a recognized risk factor for spontaneous spinal hematoma [1,2]. Spontaneous spinal subdural hematoma has been described in case reports [3,4], often presenting with diagnostic challenges similar to the case we describe. Incidental degenerative findings such as facet cysts can further influence interpretation and lead to an erroneous epidural assumption. This report describes a patient with spontaneous spinal subdural hematoma initially diagnosed as an epidural hematoma on MRI. During surgery, the dura remained tense, nonpulsatile, and bluish after epidural decompression, prompting durotomy and confirming a subdural clot. The primary aim is to highlight this intraoperative appearance as a practical clue when imaging is equivocal, particularly in anticoagulated patients.

## Case presentation

**Figure 1:**
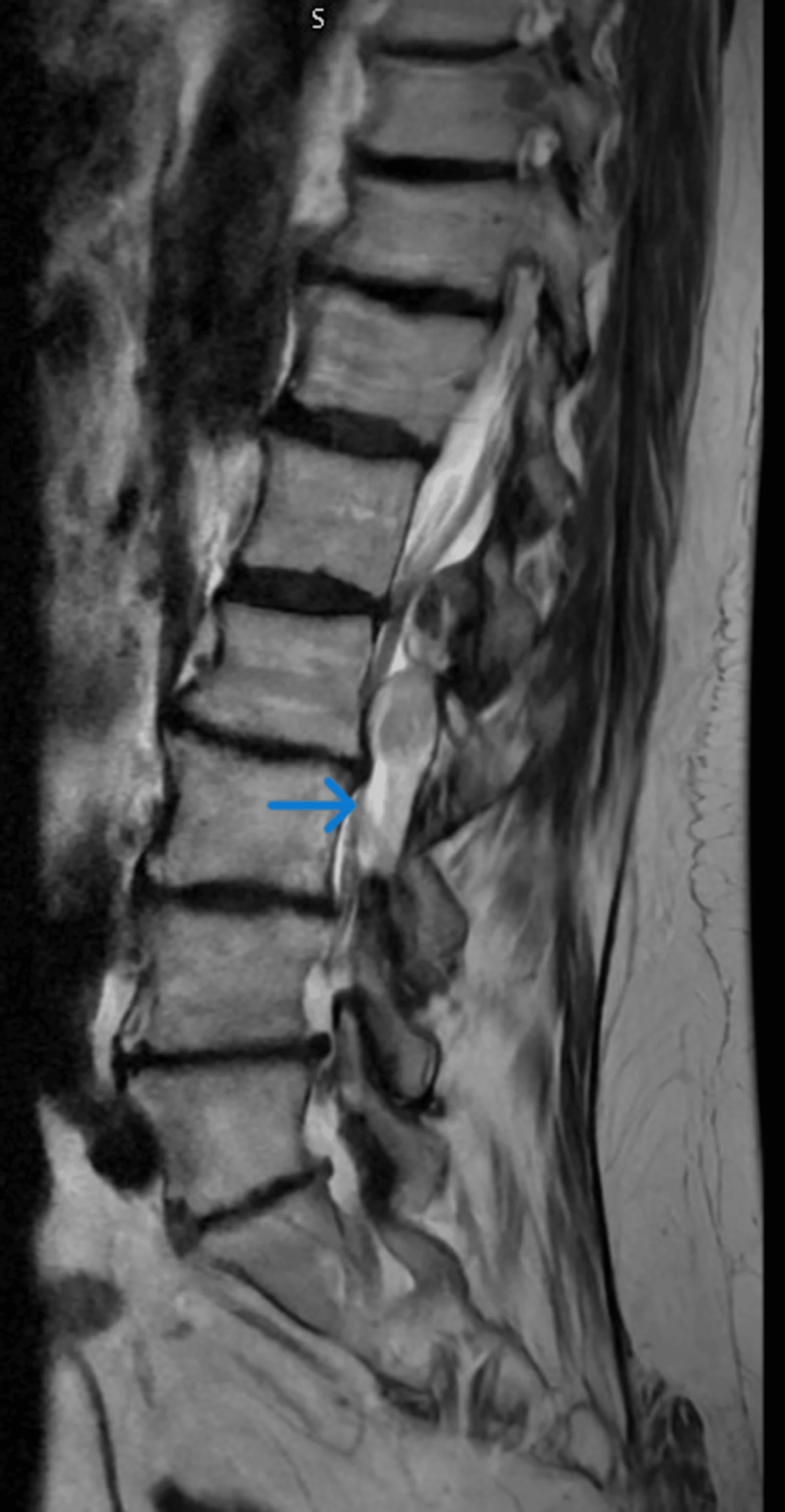
Sagittal T2-weighted MRI of the lumbar spine Sagittal T2‑weighted MRI demonstrating a dorsal hyperintense collection from L1 to L3 compressing the thecal sac (arrow).

**Figure 2:**
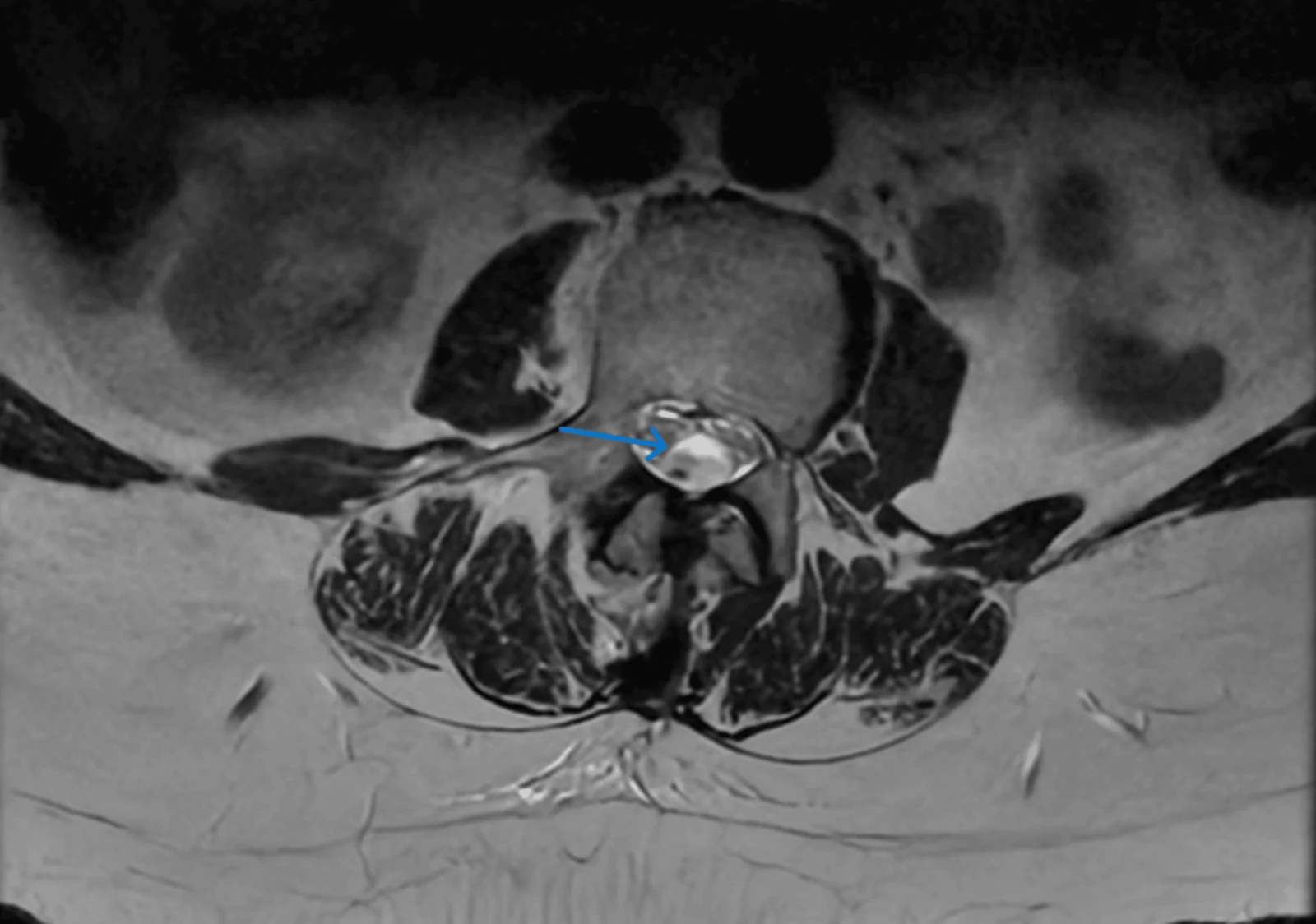
Axial T2‑weighted MRI at L3 Axial T2‑weighted MRI at L3 showing ventral displacement of the cauda equina caused by the dorsal collection (arrow).

A 73‑year‑old woman receiving apixaban for recurrent pulmonary embolism developed sudden lumbar pain radiating to both legs. By the next morning, she had right‑sided leg weakness and urinary retention. MRI showed a long dorsal collection from L1 to L3 that was reported as an epidural hematoma (Figures 1, 2).

A right L1-2 facet joint cyst was also present, which supported the initial epidural interpretation.

Urgent decompression was performed about nine hours after the onset of weakness and urinary retention through an L1-L4 laminectomy. The facet cyst was removed along with a small adjacent epidural hematoma. A facet cyst usually appears as a chronic, focal, extra‑axial mass with a firm, yellow appearance during surgery. In this case, it acted as a radiological distractor. A facet cyst does not cause a tense or bluish dura, and it does not produce loss of dural pulsatility. Its presence should not prevent consideration of a subdural source when the appearance of the dura does not match the imaging report.

**Figure 3:**
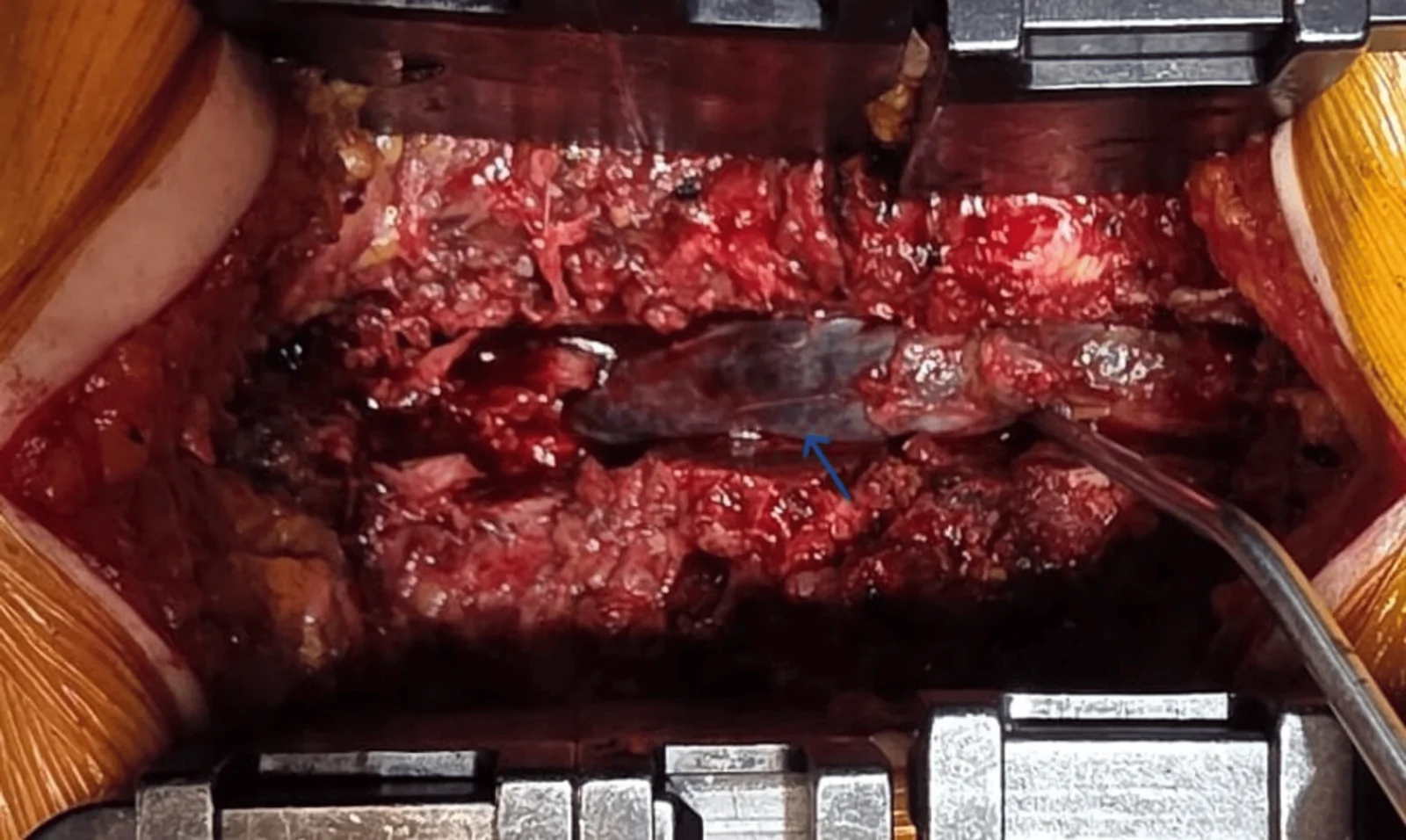
Intraoperative view of the dural sac after laminectomy Intraoperative photograph showing tense bluish discoloration of the dural sac after L1–L4 laminectomy (arrow).

Despite adequate epidural decompression, the dural sac remained firm, nonpulsatile, and bluish (Figure 3).

This appearance prompted a midline durotomy. The arachnoid was intact, and a dark, organizing subdural hematoma was found. The clot was removed, and the cauda equina was inspected to confirm decompression. The dura was closed with 5‑0 Prolene sutures and reinforced with fibrin sealant.

**Figure 4:**
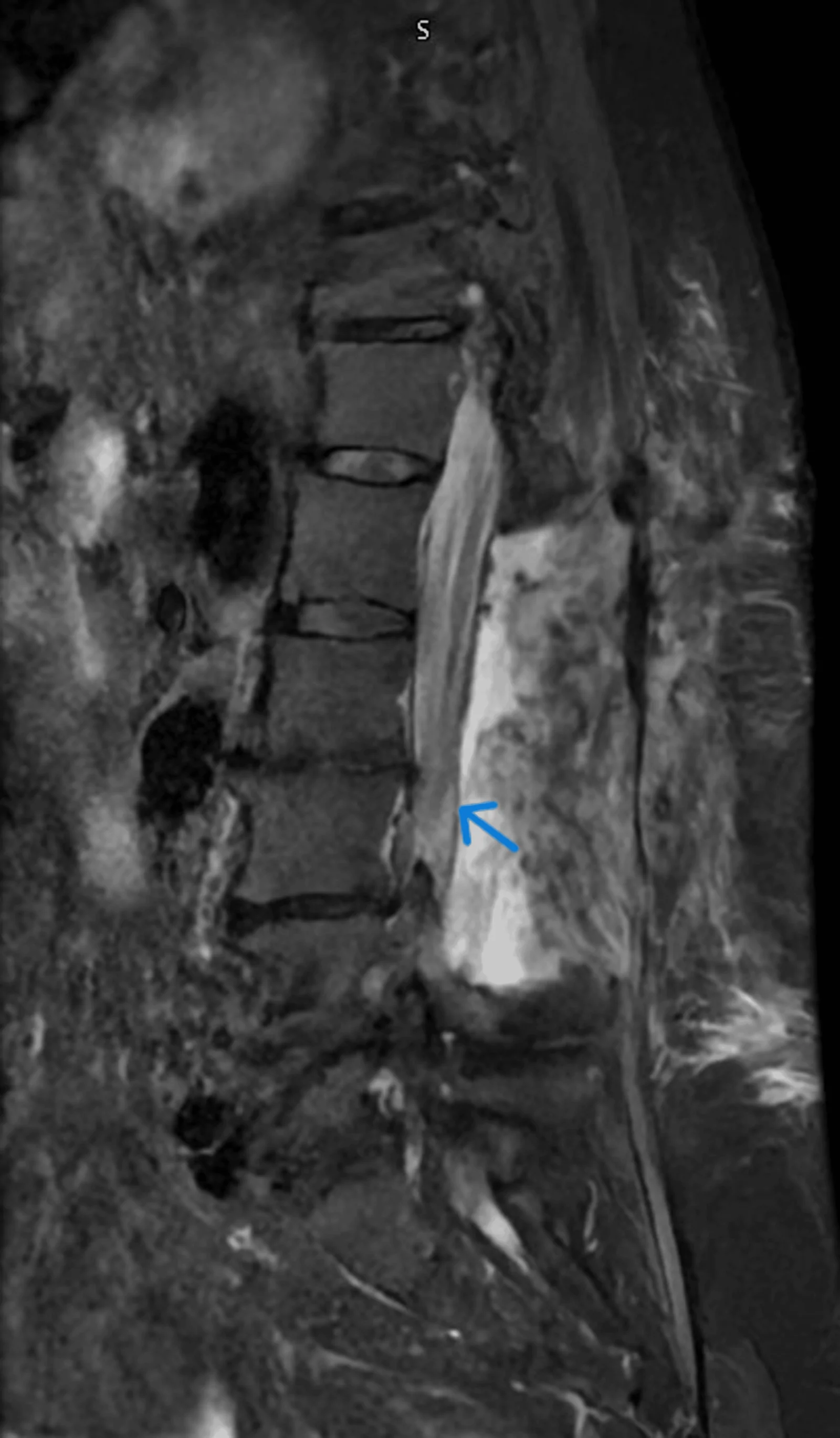
Postoperative sagittal T2‑weighted MRI of the lumbar spine Postoperative sagittal T2‑weighted MRI at two weeks demonstrating complete evacuation of the subdural hematoma and restoration of thecal sac contour (arrow).

Radicular pain resolved immediately. At two weeks, the left leg had recovered fully, and the right leg showed partial motor improvement. Apixaban was restarted at two weeks after MRI confirmed complete decompression (Figure 4).

At three months, she was walking independently with full neurological recovery.

## Discussion

Spontaneous spinal subdural hematoma is an uncommon cause of acute neurological deterioration and represents a small proportion of intraspinal hemorrhages compared with epidural hematoma [1,2]. The underlying mechanism remains uncertain; proposed explanations include rupture of fragile subdural bridging veins or extension of bleeding from adjacent compartments [3]. The condition has been described in a range of clinical settings, including idiopathic cases, post-traumatic cases, and in patients on anticoagulation [4-7]. Among these, several reports specifically describe spinal subdural hematoma in patients taking apixaban, even when coagulation parameters are normal [5,6]. This indicates that normal laboratory values do not exclude clinically significant hemorrhage in patients on direct oral anticoagulants [2]. Cases with atypical systemic responses, such as those presenting with Takotsubo cardiomyopathy, have also been reported, supporting the broad clinical spectrum of this condition [8].

Distinguishing spinal subdural hematoma from epidural hematoma on MRI is difficult because both may appear as crescentic or biconvex dorsal collections and both can displace the thecal sac. Epidural hematoma often produces focal, asymmetric compression, whereas subdural hematoma may create a more diffuse or circumferential pattern [1,3]. In this case, the presence of a right L1-2 facet cyst and coronal scoliosis acted as diagnostic distractors, reinforcing an epidural interpretation despite the subdural origin.

Dural discoloration may also arise from venous engorgement or patient positioning, as described by Kreppel et al. [2]; however, in the present case it persisted after complete epidural decompression and was accompanied by loss of pulsatility, distinguishing it from these benign confounders. In our review of the literature, similar descriptions, such as a violaceous dura, persistent thecal sac tension, or minimal epidural clot despite marked compression, were noted in cases where imaging was misleading (Table 1).

**Table 1:** Reported cases of spontaneous spinal subdural hematoma with diagnostic or intraoperative relevance Summary of published cases describing spontaneous spinal subdural hematoma with diagnostic or intraoperative relevance, including age, anticoagulant status, affected level, initial MRI interpretation, diagnostic confounders, intraoperative dural appearance, the specific intraoperative clue prompting durotomy, and reported neurological outcomes. 'Not applicable' reflects cases in which no durotomy or intraoperative inspection occurred, whereas 'Not reported' indicates that the original source did not document the relevant detail. Abbreviation: SSDH, spinal subdural hematoma.

Author (Year)	Age/Sex	Anticoagulant	Level	MRI Diagnosis	Diagnostic Confounder(s)	Intraoperative Dural Appearance	Clue Prompting Durotomy	Outcome (as reported)
Faiek (2020) [6]	84/M	Apixaban	T12-L3	SSDH	Not reported	Not reported	Imaging consistent with SSDH	Incomplete recovery (Residual paraparesis at 4 months)
Mchaourab (2019) [5]	68/M	Apixaban	T1-T5, L4-S1	SSDH	MRI initially reported as Syringomyelia	Not applicable	Not applicable	Incomplete recovery (residual bladder dysfunction, assisted mobilization)
Zari (2025) [4]	34/F	None	C7-T2	SSDH	Not reported	Not reported	Imaging consistent with SSDH	Incomplete recovery (Improved motor function)
Andour (2023) [7]	45/M	None	L1- S2	SSDH	Traumatic context with cerebral edema	Not applicable	Not applicable	Radiological resolution; clinical improvement

This suggests that although the tense bluish dura sign is not entirely novel, it appears underreported in the literature and may represent a clinically relevant intraoperative checkpoint when MRI findings are equivocal.

Urgent surgical decompression remains the recommended management for progressive neurological deficits [2,9]. In this patient, decompression occurred approximately nine hours after symptom onset. Pain and bladder dysfunction resolved, and motor recovery was substantial, although asymmetry persisted, likely reflecting the predominantly right‑sided hematoma and the coexisting facet cyst producing focal nerve root compression.

Management of direct oral anticoagulants (DOACs) requires careful timing. Perioperative anticoagulation decisions were made in consultation with the hematology service. Their recommendations followed general principles used in urgent neuraxial bleeding: holding the DOAC, reserving reversal for unstable cases, and restarting anticoagulation only after confirmed hemostasis. Accordingly, no reversal agent was used because the interval since the last apixaban dose and normal coagulation studies suggested a manageable bleeding risk. Mechanical prophylaxis was initiated postoperatively, followed by tinzaparin at 72 hours and full-dose apixaban at two weeks, after MRI confirmed complete decompression. This approach balanced thromboembolic risk with postoperative bleeding risk in accordance with established perioperative DOAC guidance [10]. We acknowledge that this is a single case report, and the “tense bluish dura” sign should be interpreted as an intraoperative clue rather than a validated diagnostic sign.

## Conclusions

When a standard lumbar laminectomy for a presumed epidural hematoma fails to restore dural pulsatility and reveals a firm, cyanotic dural sac, the operating surgeon should broaden the differential to include subdural hematoma. Failure to consider dural exploration in this setting may result in incomplete decompression and persistent cauda equina compression. This “tense bluish dura” sign may serve as a useful intraoperative checkpoint that should prompt consideration of dural exploration, especially in anticoagulated patients with coexisting degenerative distractors on MRI.
